# Tsp-1^+^ microglia attenuate retinal neovascularization by maintaining the expression of Smad3 in endothelial cells through exosomes with decreased miR-27a-5p

**DOI:** 10.7150/thno.84236

**Published:** 2023-06-26

**Authors:** Qian Luo, Zihua Jiang, Jingyi Jiang, Linxi Wan, Yan Li, Yuke Huang, Jin Qiu, Keming Yu, Jing Zhuang

**Affiliations:** 1 State Key Laboratory of Ophthalmology, Zhongshan Ophthalmic Center, Sun Yat-sen University, No.7 Jinsui Road, Tianhe District, Guangzhou, 510060, China.; 2 Guangdong Provincial Key Laboratory of Ophthalmology and Visual Science, Guangzhou, 510060, China.

**Keywords:** Oxygen-induced retinopathy, Single-cell RNA-sequencing, microglia, exosomes, Tsp-1.

## Abstract

**Rationale:** Microglia with a repertoire of functions are critical in pathological regulation of angiogenesis in the retina. However, retinal microglia with beneficial contributions and corresponding mechanisms during pathological neovascularization are poorly understood.

**Methods:** We conducted a bioinformatic comparison of public single-cell RNA transcriptome data between retinal microglia from mice with oxygen-induced retinopathy (OIR) and an antiangiogenic microglial population named MG3 from the spine. The essential beneficial factor thrombospondin-1 (Tsp-1) from microglia was discovered and then validated in the retina of mice with OIR at P17. Exosomes were isolated from Tsp-1-knockout microglia (KO-Exos) and Tsp-1^+^ microglia (NT-Exos). Human umbilical vein endothelial cells (HUVEC) morphology studies, exosomes' miRNA sequencing, luciferase reporter assay, miRNA loss of function studies, and intravitreal injection were used to explore the mechanism of Tsp-1 and microglia-associated retinal angiogenesis.

**Results:** The bioinformatic analyses of single-cell RNA-seq data indicated that a subtype of retinal microglia named RMG1 shares features with MG3 in regulating wound healing, cell adhesion, and angiogenesis. Remarkably, Tsp-1, an extracellular matrix protein with robust inhibition of angiogenesis, was especially expressed in both MG3 and RMG1. However, the scarcity of Tsp-1^+^ cells was observed in RMG1, which could be an obstacle to attenuating retinal neovascularization. Subsequently, we found that exosomes derived from Tsp-1^+^ microglia inhibit the migration and tube formation of HUVEC. Moreover, the knockout of Tsp-1 led to the enrichment of miR-27a-5p in exosomes from microglia and promoted angiogenesis compared to that of NT-Exos* in vitro*. Furthermore, in the luciferase reporter assay on the transcriptional activity of the promoter, we demonstrated that Tsp-1 negatively regulates miR-27a-5p expression. In addition, SMAD family member 3 (Smad3), a receptor-activated Smad protein that is conducive to vascular homeostasis, was defined as a functional target gene of miR-27a-5p. These data were consistently confirmed *in vivo* in the retina of mice with OIR.

**Conclusion:** Collectively, the Tsp-1/miR-27a-5p/Smad3 axis is involved in microglia-related and exosome-mediated antiangiogenic regulation of the retina. Therefore, this study reveals a novel mechanism by which retinal microglia maintain vascular homeostasis, thereby providing a new therapeutic target for pathological neovascularization.

## Introduction

Pathologic angiogenesis in the retina is a typical feature of major blindness-causing diseases 1-3, including diabetic retinopathy (DR), retinopathy of prematurity (ROP), and age-related macular degeneration (AMD). The irregular sprouting and growth of new capillaries from the retinal vascular beds can result in irreversible vision loss, particularly when accompanied by increased vascular permeability. The process of pathological neovascularization in ocular diseases is multifaceted and influenced by numerous factors. Certain pivotal regulators of this process have been employed in the development of therapeutic strategies aimed at globally inhibiting neovascularization in ocular tissues, such as anti-VEGF drugs. However, the broad inhibition of VEGF may affect retinal and blood vessel homeostasis, compromising the health of normal blood vessels as well as neurons and glia in the retina 4-6. The ideal therapy should be capable of precisely attenuating abnormal blood vessel growth while ensuring the healthy process of revascularization. Moreover, the precise and specific mechanism of ocular angiogenesis remains poorly defined due to the complex cellular composition and heterogeneity within the retinal microenvironment.

Microglia have been recognized as special immune system components that mainly exist in the central nervous system, as well as the retina 7. These cells have been extensively studied because of their repertoire of roles in the immune response, neuroprotection, and phagocytosis of cellular debris 8-13. Recently, numerous studies have noted the multiple roles of microglia in angiogenic regulation. Emerging studies with single-cell RNA sequencing (scRNA-seq) technology have revealed the harmful roles of most retinal microglia in the progression of some vascularization-related diseases 14-16. Many of these studies have noted that a large group of microglia promote retinal pathological angiogenesis. These microglia emerge during the OIR process and develop characteristics such as proliferative, hypermetabolic, and even necrotic characteristics, which ultimately lead to a proangiogenic phenotype. However, in some other areas of the central nervous system, the situation is different. A recent study reported that some microglia in the neonatal mouse spinal cord transiently appeared around the lesion during injury treatment, which supports a microenvironment for axon regrowth via auxiliary regulation of endothelial wound healing 17. In general, the high cell diversity of microglia in angiogenic regulation deserves to be investigated. Moreover, it remains to be determined whether a group of retinal microglia has beneficial effects during pathological angiogenesis. Furthermore, the concrete details of cellular interactions, either by physical contact or secretion, between microglia and vascular endothelial cells (ECs) are largely unknown.

Due to their high mobility, microglia are capable of migrating to a specific microenvironment, where they can physically attach to ECs and subsequently transfer regulatory signals 18,19. Exosomes, as crucial mediators of intercellular communication and signal transduction in paracrine signalling, might play an important role in the transfer process. Exosomes are cell-derived nanovesicles with principal components, including proteins, lipids, and nucleic acids. Endocytosis of exosomes changes the metabolism and behaviour of recipient cells, for example, via exosomal microRNA-induced mRNA degradation 20,21. Previous studies have demonstrated that microglia strongly secrete exosomes 22,23. In a particular polarization state, microglia produce exosomes to promote angiogenesis in the central nervous system 24. Nevertheless, since some microglia might play an antiangiogenic role, exploration of the underlying antiangiogenic mechanisms mediated by exosomes in particular microglial subpopulations is urgently needed.

In this study, we utilized bioinformatic tools and public datasets to analyse the single-cell transcriptome characteristics of microglia from the spinal cord and retina. For regulation of angiogenesis, we found more antiangiogenic features in MG3 microglia, a microglial population from the spine, compared to a similar microglial group, RMG1, from the retina. Moreover, Tsp-1, an extracellular matrix protein that potently inhibits angiogenesis, critically contributes to the distinction of these two groups of microglia. To confirm the bioinformatic results, we utilized an OIR animal model and the BV2 microglial cell line to investigate the role and function of Tsp-1^+^ microglia in the retinal microenvironment. A series of studies of Tsp-1 gene loss-of-function, exosome experiments of microglia, and morphological tests of ECs were conducted to reveal the underlying mechanism of retinal angiogenic regulation via intercellular communication between microglia and ECs. Our data indicated that exosomes derived from Tsp-1^+^ microglia strongly inhibited the migration of ECs. miR-27a-5p is highly related to the function of Tsp-1^+^ microglial exosomes. Smad3 of the recipient ECs is an essential target gene that responds to miR-27a-5p in microglia-derived exosomes. Thus, our discoveries provide new insights into aberrant retinal angiogenesis.

## Materials and methods

### Bioinformatic analysis of scRNA-seq datasets

R (version 4.2.2) was used for analysis. The scRNA-seq data of spine microglia, GSE150871, consisting of five independent samples from mouse spinal cord repair, were downloaded from the Gene Expression Omnibus. Quality control was implemented in Seurat (version 4.3). For each dataset, genes expressed in fewer than five cells were removed, and then, cells with more than 2,000 features, with a lower than 80,000 RNA count, and that expressed less than 15% mitochondrial genes were used. Then, the five datasets were integrated by the IntegrateData function to remove batch effects. The merged data were normalized by the LogNormalize method of the NormalizeData function with a scale factor of 10,000. The FindVariableFeatures function was used to select the top 5,000 feature genes. Data were scaled with mitochondrial percent by the ScaleData function. Principal component analysis (PCA) and t-distributed stochastic neighbour embedding (t-SNE) were based on the top 5,000 variable features. Clustering results were visualized by t-SNE plots with the top 14 PCs. Differentially expressed genes of each cluster were analysed by the FindAllMarkers function. The cell type of each cluster was identified by the marker genes provided by Yi Li 17. All microglial cells were selected to cluster and identify subpopulations with a similar method after removing the cell cycle effect by the CellCycleScoring function. Differentially expressed genes were analysed again by the FindAllMarkers function. The ClusterProfile (version 4.4.4) package was used for Gene Ontology (GO) enrichment analysis of MG3 with the top 100 feature genes ranked by log-scaled fold change (FC) and with an adjusted P value less than 0.01.

For the scRNA-seq data of the OIR retinal microglia, GSE150703, with Normoxia and OIR retina samples from P14 and P17 mice, was downloaded from Gene Expression Omnibus. Similarly, genes expressed in fewer than five cells were removed, and cells with more than 100 features that expressed less than 10% mitochondrial genes were used. Then, the datasets were integrated, normalized, featured, scaled, and clustered with the same methods mentioned above. The clustering results were visualized by t-SNE plots with the top 15 PCs. All microglial cells were identified by Aif1 and Csf1r and selected to cluster and identify subpopulations with a similar method.

### Mice and OIR model

C57BL/6J mice were purchased from the Animal Laboratories of Zhongshan Ophthalmic Center (Guangzhou, China). All animal studies complied with the Association for Research in Vision and Ophthalmology Statement for the Use of Animals in Ophthalmic and Vision Research and were approved by the Institutional Animal Care and Use Committee of Zhongshan Ophthalmic Center (IACUC no. O2022002). All animals were housed in a pathogen-free environment with regulated photoperiods (12 h light/12 h darkness).

The OIR model was established using a previously described method 25. Briefly, mouse pups with nursing mothers were exposed to 75% O_2_ from P7 to P12 in an Oxy Cycler system (BioSpherix, NY, USA) to induce retinal vaso-obliteration (VO) and then returned to room air (RA, 21% O_2_) to induce retinal neovascularization (NV), which reached its peak at P17. Age-matched mice kept in RA served as the Normoxia controls. At P17, mice were sacrificed by CO_2_ euthanasia, and eyes were enucleated for further investigation.

### Retinal cryosection staining and immunofluorescence assay

Eyes were removed, enucleated, and embedded in optimal cutting temperature compound (OCT, Tissue-Tek, CA, USA) at -80 °C overnight, and 10-μm serial cryosections were prepared. An immunofluorescence assay was performed with standard protocols. The retina sections were blocked with 5% bovine serum albumin (BSA) in phosphate-buffered saline (PBS) for 30 min at room temperature and then incubated with primary antibodies overnight at 4 °C, followed by incubation with secondary antibodies for 1 h at 37 °C in the dark. Double staining of Iba-1 and Tsp-1 was first stained with rabbit anti-Iba-1 (1:250; cat. no. ab178846, Abcam, MA, USA) and mouse anti-Thrombospondin 1 (1:250; cat. no. ab1823, Abcam, MA, USA), and then incubated with Alexa Fluor 488 anti-rabbit IgG (1:500; cat. no. 4412, Cell Signaling Technology, MA, USA) and Alexa Fluor 555 anti-mouse IgG (1:500; cat. no. 4409, Cell Signaling Technology, MA, USA). Double staining of Isolectin B4 (IB4) and Smad3 was first stained with rabbit anti-Smad3 (1:200; cat. no. A19115, Abclonal, Wuhan, China) and Isolectin B4 (1:500; cat. no. I21411; B4-488, Invitrogen, CA, USA), and then incubated with Alexa Fluor 555 anti-rabbit IgG (1:500; cat. no. 4413, Cell Signaling Technology, MA, USA). The staining of IB4 was only incubated with Isolectin B4 (1:500, cat. no. I121411; B4-488, Invitrogen, CA, USA) overnight. Afterward, the Nuclei were stained with DAPI for 5 min in the dark. Immunofluorescence-stained samples were observed using a confocal microscope (Zeiss LSM 980 with Airyscan 2, Oberkochen, Germany).

### Cell culture

BV2 microglial cell line and HEK-293T cell line were purchased from the National Infrastructure of Cell Line Resource (Beijing, China) and cultured in DMEM (Gibco, CA, USA) supplemented with 10% fetal bovine serum (FBS). HUVEC were purchased from ScienCell and cultured in ECM (ScienCell, CA, USA) with 5% FBS and 1% endothelial cell growth supplement (ECGS). The cells were maintained at 37 °C in a humidified atmosphere with 5% CO_2_.

### CRISPR/Cas9 knockout Tsp-1 in BV2 cell lines

To knock out the Tsp-1 gene, we designed two single-guided RNAs (sgRNA 1: Forward 5′- CCA GGG TGT CGA ACA TGC CA-3′ Reverse: 5′- TGG CAT GTT CGA CAC CCT GG -3′; sgRNA 2: Forward 5′- GAC CTC AGC CTG ACC GTC CA -3′ Reverse: 5′- TGG ACG GTC AGG CTG AGG TC -3′) and cloned the target sequences into the lentiCRISPRv2 vector (Addgene). Lentivirus for Tsp-1 sgRNA and vector control were generated in HEK-293T cells using standard lenti-packaging vectors. After infecting BV2 cells with the lentivirus for 48 h and selecting them with puromycin (2 μg/mL) for 10 days, the target monoclonal stable cell lines were obtained. Tsp-1 deletion in individual monoclonal cell lines was further verified by DNA sequencing, western blot, and immunofluorescence.

### Western blot

Mouse retinal tissues, cells, and exosomes were lysed with RIPA buffer (Beyotime, Jiangsu, China) containing 1% Phenylmethanesulfonyl fluoride (PMSF). Western blotting was performed according to standard protocols. The primary antibodies used were as follows: rabbit anti-β-Tubulin (1:10,000; cat. no. 10094-1-AP; ProteinTech, IL, USA), mouse anti-GAPDH (1:10,000; cat. no. M00227-2; Boster Biological Technology, CA, USA), rabbit anti-CD63 (1:1,000; cat. no. EXOAB-CD63A-1; Systems Biosciences, CA, USA), rabbit anti-TSG101 (1:1,000; cat. no. ab125011; Abcam, MA, USA), rabbit anti-GRP94 (1:1,000; cat. no. 20292, Cell Signaling Technology, MA, USA), rabbit anti-Thrombospondin 1 (1:1,000; cat. no. BA2130-2; Boster Biological Technology, CA, USA), and rabbit anti-Smad3 (1:1,000; cat. no. 5678, Cell Signaling Technology, MA, USA). The secondary antibodies used were HRP-conjugated anti-mouse IgG (1:3,000; cat. no. 7076s; Cell Signaling Technology, MA, USA) or anti-rabbit IgG (1:10,000; cat. no. 7074s; Cell Signaling Technology, MA, USA). GAPDH or β-Tubulin served as a loading control. The bands were visualized using the ECL chemiluminescence system (ChemiDoc MP Imaging System, BIO-RAD, CA, USA). FIJI (ImageJ) software was used for quantification.

### Immunofluorescence cell staining

We captured cells on the cover glass and fixed them with 4% paraformaldehyde (PFA, Invitrogen, CA, USA) for 15 min. The cells were incubated with primary antibodies overnight at 4 °C, followed by incubation with secondary antibodies that were fluorescently labelled. The primary antibodies used were as follows: rabbit anti-VE-cadherin (1:250; cat. no. 2500, Cell Signaling Technology, MA, USA), rabbit anti-Iba-1 (1:250; cat. no. ab178846, Abcam, MA, USA), and mouse anti-Thrombospondin 1 (1:250; cat. no. ab1823, Abcam, MA, USA). The secondary antibodies used were Alexa Fluor 488 anti-rabbit IgG (1:500; cat. no. 4412, Cell Signaling Technology, MA, USA) or Alexa Fluor 555 anti-mouse IgG (1:500; cat. no. 4409, Cell Signaling Technology, MA, USA). Afterward, the Nuclei were stained with DAPI for 5 min in the dark. All images were captured using a confocal microscope (Zeiss LSM 980 with Airyscan 2, Oberkochen, Germany) or a fluorescence microscope (Zeiss Axio Imager Z1, Oberkochen, Germany).

### Isolation, identification, and labelling of exosomes

Exosome isolation and identification followed the MISEV 2018 guidelines 26. Briefly, BV2 microglia were cultured in DMEM supplemented with 10% exosome-depleted FBS. After the cells reached 85% confluence, the exosomes were isolated from the cell-derived conditioned medium with the ultracentrifugation method 27. To remove cells, dead cells, cell debris, apoptotic bodies, and large vesicles, the collect medium was centrifuged at 300×g for 10 min, 2,000×g for 10 min, and then 10,000×g for 30 min at 4 °C. The supernatants were then ultracentrifuged at 100,000×g for 90 min at 4 °C to pellet the exosomes. The pellets were washed once with PBS and resuspended in a defined amount for further use. NanoSight NS300 (Malvern Panalytical, UK) was used to measure the concentration and size distribution of the exosomes. Transmission electron microscopy (TEM, FEI TECNAI spirit G2, OR, USA) was used to assess exosome morphology. Western blot analysis was used to verify the presence or absence of exosomal surface marker proteins, including TSG101 and CD63, and endoplasmic reticulum marker GRP94. Exosomes were labelled with a PKH26 Red Fluorescent Cell Linker Kit (PKH26, Sigma-Aldrich, MO, USA) following the manufacturer's protocol. Exosomes were added to Diluent C first, followed by incubation with PKH26 dye diluted with Diluent C (protected from light). To bind excess dye, BSA was added to the reaction system. Labelled exosomes were extracted again by ultracentrifugation and resuspended in PBS.

### Wound healing and tube formation assay

*Wound-healing assay.* A total of 4 × 10^4^ HUVEC were seeded in a 12-well plate and treated with different exosomes (0.8 μg), transfected with miR-27a-5p mimic (miR-27a-m) or synthetic mimic control (NC-m), or transfected with small interfering RNA against SMAD3 (siSMAD3) or nonspecific control siRNA (siCtrl). Then, a wound was created manually by scraping the cell monolayer with a 200-μl pipet tip. At 0 h and 24 h after the wound scratch, images of the wound spaces were captured by an inverted microscope (Nikon ECLIPSE Ts2, Tokyo, Japan) and quantitatively analyzed using FIJI (ImageJ) software. The rate of migration was determined by quantifying the total area where the HUVEC moved from the edge of the scratch to its center.

*Tube formation assay.* The matrigel matrix (Corning, NY, USA) was used to coat 96-well plates and incubated at 37 °C for at least 30 min. A total of 4×10^4^ HUVEC were seeded on the Matrigel-coated well and then treated with exosomes (0.8 μg). After 12 h, the endothelial tubule formation was observed and photographed using an inverted microscope (Nikon ECLIPSE Ts2, Tokyo, Japan). The tube formation ability was determined by measuring the number of tubule junctions and tube length using FIJI (ImageJ) software.

### Exosomal miRNA sequencing

Total RNA was extracted from the exosomes from BV2-NT and BV2-KO cell lines (NT-Exos and KO-Exos), and 50 ng RNA was used for small RNA cDNA library construction. NEBNext^®^ Multiplex Small RNA Library Prep Set for Illumina^®^ (New England BioLabs Inc., MA, USA) was used to generate sequencing libraries according to the manufacturer's instructions. Libraries were then amplified and sequenced on a HiSeq 2500 platform (Illumina, CA, USA). The experiments were performed by LC-BIO TECHNOLOGIES (Hangzhou, China).

### Reverse transcription (RT) and Real-time PCR

The total RNA of cells was isolated using TRIzol Reagent (Invitrogen, CA, USA). The total RNA of exosomes was extracted using TRIzol Reagent (Invitrogen, CA, USA) and Dr. GenTLE™ Precipitation Carrier (Takara, Dalian, China). Then, 1 μg total RNA was added to a mixed reagent for reverse transcription. Reverse transcription for miRNA was performed according to the manufacturer's instructions for the RevertAid First Strand cDNA Synthesis Kit (Invitrogen, CA, USA) or Mir-X™ miRNA First-Strand Synthesis Kit (Takara, Dalian, China). For mRNA, Total RNA was subjected to reverse transcription using a PrimeScript RT Reagent kit (Takara, Dalian, China) following the manufacturer's protocol.

The expression levels of miRNA and mRNA were measured by real-time PCR using SYBR Prime Script RT-PCR Kit on a LightCycler^®^ 480 system (Roche, Switzerland) following the manufacturer's protocol. In brief, the reactions were incubated at 95 °C for 5 min, followed by 45 cycles of 95 °C for 10 s, 60 °C for 10 s, and 72 °C for 10 s. The relative expression levels of miRNA and mRNA were quantified by the 2^-ΔΔCT^ method and normalized using U6 or Actin expression. The primers are shown in Table S1.

### Preparation of exosomes loaded with miR-27a-5p mimic/inhibitor/antagomir

To introduce miR-27a-5p mimic, inhibitor, or antagomir into exosomes, a modified method of calcium chloride transfection was used 28. 20 μg exosomes were mixed with 200 pmol miR-27a-5p mimic, inhibitor, or antagomir in PBS, and then CaCl_2_ was added (final concentration: 0.1 M). The final volume was adjusted to 300 μL using sterile PBS. The mixture was incubated on ice for 30 min followed by a heat shock at 42 °C for 1 min. Subsequently, the mixture was put back on ice for 5 min. After that, RNase A (5 μg/mL) was added to remove unloaded and free miRNAs outside the exosomes. The miR-27a-5p mimic-, inhibitor-, or antagomir-loaded exosomes were recovered with the ultracentrifugation method. The sequences of miR-27a-5p mimic, inhibitor, and antagomir are shown in Table S2.

### Luciferase reporter assay

*Promoter reporter assay.* DNA fragments from -853bp to -133bp relative to the initiation site of pre-miR-27a were inserted into pGL3-Basic-IRES (Addgene) using XhoI sites 29. These plasmids (1μg) were co-transfected with Renilla luciferase expression vector (pRL-CMV) (10 ng) to BV2-NT or BV2-KO cells grown in 24-well plates using Lipofectamine^®^ 3000 Transfection Reagent (Invitrogen, CA, USA). Luciferase activity assays were performed after 48 h of transfection using the Dual-Luciferase^®^ Reporter Assay System (Promega, WI, USA) according to the manufacturer's instructions. The ratio of Firefly luciferase activity to Renilla luciferase activity provides relative luciferase activity.

*3'UTR reporter assay.* Partial segments of the 3'UTR of Smad3 mRNA containing the predicted miR-27a-5p-binding sequences were inserted between the HindIII and SpeI restriction enzyme sites in the pMIR-report vector (Addgene). The luciferase reporter vector (pMIR-report-Smad3-WT, WT) and its substitution mutant (pMIR-report-Smad3-Mut, Mut) were constructed using the sequences in Table S3. HEK-293T cells were plated in 24-well plates and transfected with WT or Mut (1 μg), NC-m or miR-27a-m (100 nM) and pRL-CMV (10 ng). Cells were incubated for 48 h, and luciferase activity assays were detected using the Dual-Luciferase^®^ Reporter Assay System (Promega, WI, USA).

### Intravitreal injection of microglial exosomes

At P12, mouse pups received a single intravitreal (IVT) injection of microglial exosomes using a 2.5-μL Hamilton syringe with a 34-gauge needle (Hamilton, NV, USA). Specifically, 1 μL of exosomes, PKH26-labelled exosomes, and antagomir-loaded exosomes (1 μg/μL) were injected into the vitreous cavity of the left eye. Respecting IACUC guidelines, the right eyes were not injected.

### Retinal flat-mount staining

Eyes were enucleated and fixed in 4% PFA for 1 h at 4 °C. Retinas were removed carefully and incubated with Isolectin B4 (1:500, cat. no. I121411; B4-488, Invitrogen, CA, USA) overnight at 4 °C. Retinas were then rinsed in PBS and cut into four connected pieces before flat-mounted on the slides. The images were obtained using a confocal microscope (Zeiss LSM 980 with Airyscan 2, Oberkochen, Germany) or a fluorescence microscope (Leica DMi8, Wetzlar, Germany). Areas of retinal VO and NV were quantified as a percentage of total retinal areas using Adobe Photoshop software according to well-established techniques 25.

### Statistical analysis

All data were presented as mean ± SDs using Prism 9.0 software (GraphPad Prism). Data were analyzed statistically using ANOVA for comparisons of more than two groups or Student's two-tailed t-test for two-group comparisons. Results with a p < 0.05 difference were considered statistically significant.

## Results

### Bioinformatic analysis revealed a subpopulation of microglia with consistent antiangiogenic characteristics in neonatal mice with spinal cord injury and retinas of mice with OIR

**Figure 1:**
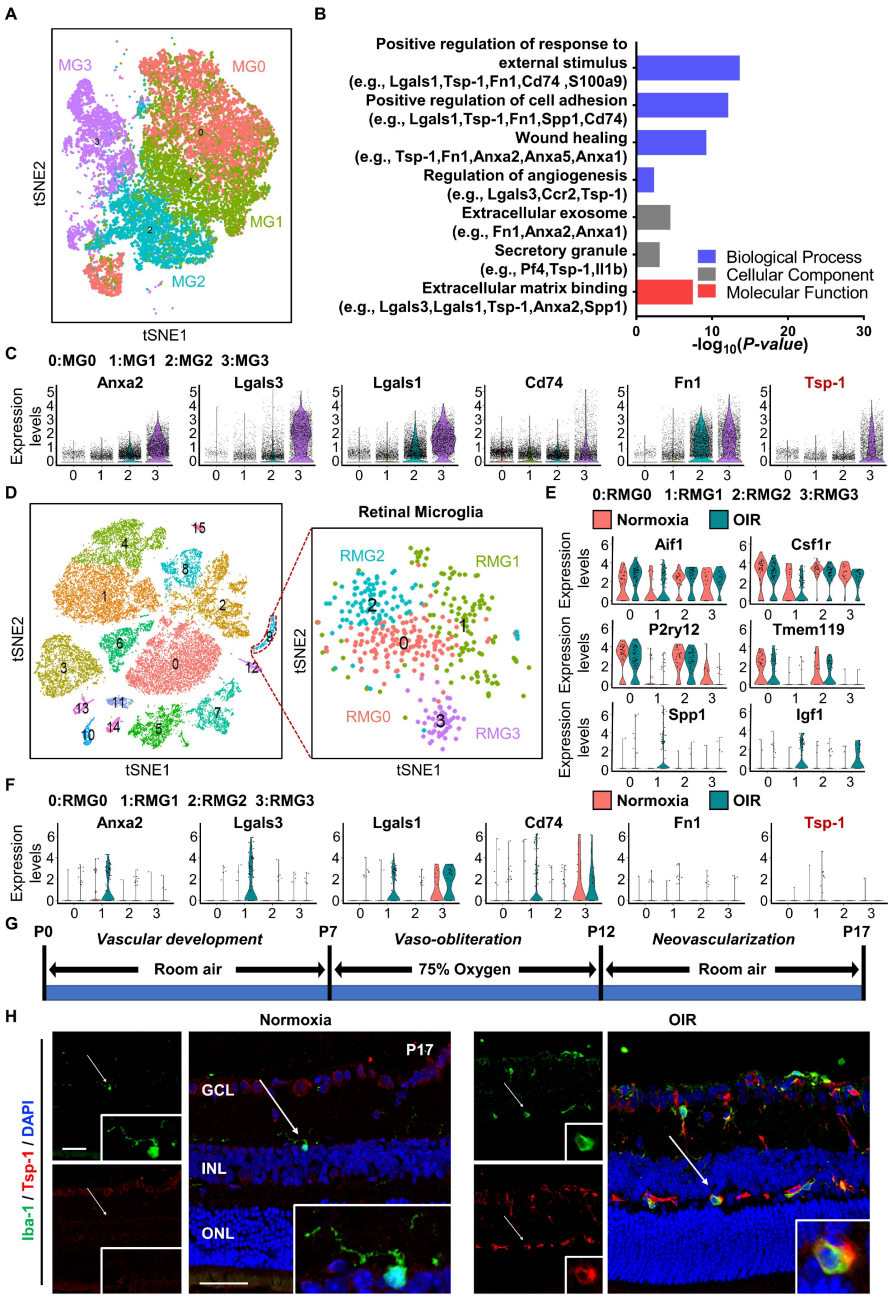
** Single-cell RNA-seq analysis revealed a subpopulation of microglia with consistent antiangiogenic characteristics in the injured spinal cord of neonatal mice and the retinas of mice with OIR. (A)** t-SNE plot showing 4 clusters of microglia isolated from the injured spinal cord of mice at postnatal Day 2. **(B)** Selected Gene Ontology (GO) terms and associated genes enriched in MG3. A two-sided statistical test in function enrichGO was used. **(C)** Violin plots showing high-level expression of Anxa2, Lgals3, Lgals1, CD74, Fn1, and Tsp-1 in MG3. Each dot represents 1 cell, and the violin represents the distribution of probability density. **(D)** t-SNE plot of cells isolated from Normoxia and oxygen-induced retinopathy (OIR) retinas at P14 and P17 (left inset) and subclustering of microglia reveal 4 distinct cell populations (right inset). Violin plots showing differential expression of **(E)** 6 microglia-related marker genes (Aif1, Csf1r, P2ry12, Tmem119, Spp1, and Igf1) and **(F)** 6 angiogenesis-related genes (Anxa2, Lgals3, Lgals1, CD74, Fn1, and Tsp-1) among the 4 clusters of retinal microglia. **(G)** Schematic illustration of the mouse OIR model. **(H)** Representative confocal images of Iba-1 and Tsp-1 expression in the Normoxia and OIR retina. In the Normoxia retina, Iba-1^+^ microglia lacked Tsp-1 expression, while Iba-1^+^ Tsp-1^+^ microglia were increased in the OIR retina. DAPI indicates the retinal layers (white arrows; Iba-1, green; Tsp-1, red; DAPI, blue). Scale bar: 50 µm. n = 8. *GCL*: ganglion cell layer, *INL*: inner nuclear layer, *ONL*: outer nuclear layer.

To gain insight into the angiogenic regulation mediated by microglia, we analysed the single-cell RNA sequencing dataset GSE150871, which contains transcriptomic information of single cells isolated from the lesion of neonatal mouse spinal cord, followed by flow cytometry sorting with CD11b^+^CD45^low^ gating. After filtering, 27,429 cells in total were obtained from the lesion tissue at different time points. Fourteen clusters were generated from unsupervised clustering and labelled with given marker genes. Clusters with high expression of the microglial cell markers Aif1 and Csf1r 30,31 were selected (n = 23,192 cells) and reclustered into four distinct groups (MG0-MG3) after removal of cell cycle effects (Figure 1A). MG3 has been validated to express activated microglial makers such as Spp1 and Igf1, and functionally, these cells strongly contribute to the healing of spinal cord injury with high expression of proteinase inhibitors and fibronectin 17. Since vascular regulation is also important for the healing process, we focused on vascular-associated features of MG3. As expected, Gene Ontology (GO) analysis indicated that many pathways related to neovascularization were statistically significant (Figure 1B). Marker genes of MG3 were significantly enriched in wound healing, angiogenesis, and positive regulation of cell adhesion, including Anxa2, Lgals3, Lgals1, Cd74, Fn1, and Tsp-1 (Figure 1C). Notably, Tsp-1, an important inhibitory factor of neovascularization 32, was highly expressed in MG3, which indicates a potential functional role of MG3 in antiangiogenesis.

The retina is a part of the central nervous system and frequently suffers from some diseases characterized by pathological angiogenesis. Moulding the antiangiogenic ability from MG3 to retinal microglia may have significant effects. We focused on the oxygen-induced retinopathy (OIR) mouse model, a clinically relevant animal model of retinal neovascularization, to search for an MG3-like retinal microglial population during pathological angiogenesis. We analysed a single-cell RNA sequencing dataset GSE150703, which provides the single-cell atlas of retinas of mice with OIR at P14 and P17 14. With quality control, 31,271 cells of the whole retina were obtained, and sixteen clusters were generated through unsupervised clustering. Clusters with Aif1 and Csf1r markers were further separated as retinal microglia (n = 450 cells) and reclustered into four distinct clusters (RMG0-RMG3) (Figure 1D). Specifically, as shown in Figure 1E, RMG0 and RMG2 express genes such as P2ry12 and Tmem119 associated with microglial homeostasis, while RMG1 and RMG3 express activated microglial markers such as Spp1 and Igf1. More importantly, RMG1 was significantly enriched in the OIR group. Like MG3, Anxa2, Lgals3, Lgals1, and Cd74 were upregulated in the OIR group of RMG1 (Figure 1C, 1F). Furthermore, there was a small group of microglia expressing Fn1 and Tsp-1 in RMG1 with a slightly increasing number but a limited absolute ratio (Figure 1F). Together, these results indicate that hypoxia-induced RMG1 microglia have the potential to regulate angiogenesis, similar to MG3 microglia induced by spinal cord injury. However, compared to that of MG3, the antiangiogenic potential of RMG1 might not be fully realized due to the limited quantity of microglia in the retina and the relative lack of expression of the robust antiangiogenic factor Tsp-1. Thus, the decrease in activated Tsp-1^+^ retinal microglia might have a major impact on the regulation of retinal angiogenesis.

To test this hypothesis, we adopted the OIR model and experimentally validated the expression pattern of Tsp-1 in activated microglia from the retina at P17 (Figure 1G). Immunofluorescence staining indicated that only a few induction of brown adipocytes 1 (Iba-1)^+^ microglia existed and showed a ramified-resting state featuring small cell bodies with thin and long processes in the Normoxia retina. In contrast, Iba-1^+^ microglia were significantly increased in the OIR retina. In particular, most microglia exhibited an activated state characterized by enlarged cell somas with thick and short lamellipodia. Moreover, several cells with robust Tsp-1 signal colocalized with Iba-1^+^ were observed in the OIR retina (Figure 1H). Taken together, these results indicate that a type of Tsp-1^+^ microglia particularly exists in the OIR retina and potentially responds to neovascularization.

### Tsp-1 inhibits HUVEC migration and tube formation through exosomes derived from microglia

**Figure 2:**
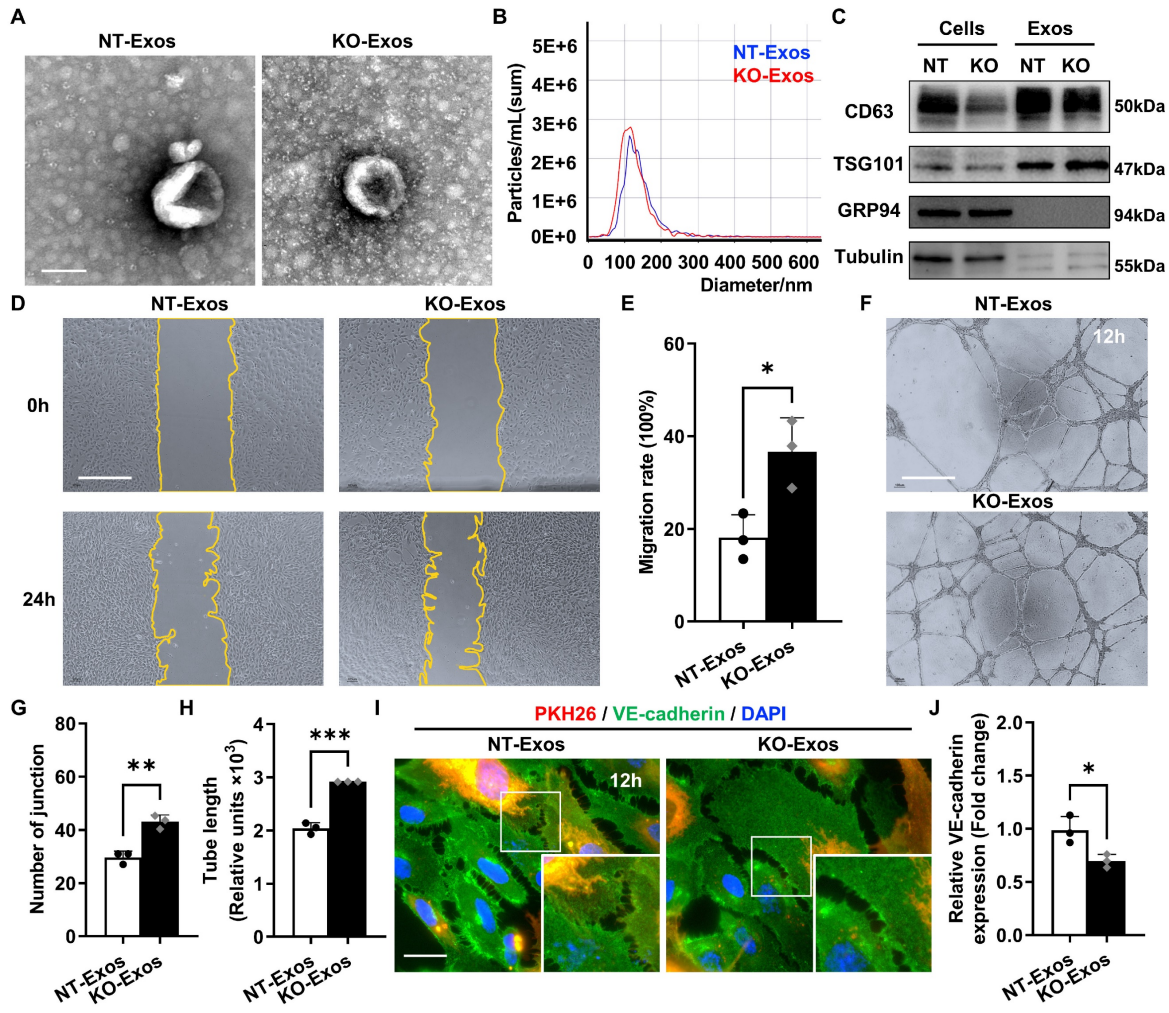
**Exosomes derived from Tsp-1 KO or NT microglial cell lines demonstrate different influences on the migration, tube formation, and intercellular junctions of HUVEC. (A)** Representative TEM images of NT-Exos and KO-Exos. The diameter of the vesicles was approximately 50-150 nm. Scale bar: 100 nm. n = 3. **(B)** NTA indicated the average size and number of exosomes (NT-Exos: 97.9% of exosomes had a size of 117.4 nm, blue; KO-Exos: 96.6% of exosomes had a size of 110.9 nm, red). n = 3. **(C)** Western blot analysis of exosomal protein markers in cell lysates or exosomes showed the presence of CD63 and TSG101 and the absence of GRP94 in NT-Exos and KO-Exos. β-Tubulin was included as a loading control. n = 3. Representative images **(D)** and quantification of the wound-healing assay **(E)** in HUVEC after treatment with NT-Exos or KO-Exos at 0 h and 24 h post-scratch. There was a significant decrease in cell migration rates in the presence of NT-Exos compared with KO-Exos. Scale bar: 500 μm. *P < 0.05. n = 3. Representative images **(F)** and quantification of tube formation, including the number of tubule junctions **(G)** and tube length **(H)**, in HUVEC treated with NT-Exos or KO-Exos showed decreased HUVEC tubule connections in the NT-Exos-treated HUVEC compared to KO-Exos group. Scale bar: 500 μm. ***P < 0.001, **P < 0.01. n = 3. Representative images **(I)** and statistical analysis **(J)** of vascular endothelial cadherin (VE-cadherin) staining in the NT-Exos- or KO-Exos- treated HUVEC. Exosomes were labelled with PKH26. There was a prominent elevation of VE-cadherin expression at intercellular junctions in the NT-Exos-treated HUVEC compared to the KO-Exos group. (white box; PKH26, red; VE-cadherin, green; DAPI, blue). Scale bar: 20 μm. *P < 0.05. n = 3. Results are expressed as mean ± SD.

To reveal the bioactivity of Tsp-1 in some activated microglia, we used BV2 microglial cell line, a well-characterized and extensively employed model, in our study. Co-staining of Iba-1 and Tsp-1 also indicated that BV2 microglia could be a valid substitute for Tsp-1^+^ microglia in the OIR retina to some extent. Moreover, we generated a Tsp-1 knockout monoclonal cell line (BV2-KO) via the lentiviral vector lentiCRISPRv2 (Figure S1) and a corresponding control cell line via a negative target vector (BV2-NT). Given the bioactivity of exosomes as crucial mediators of intercellular communication and signal transduction, we isolated exosomes derived from BV2-NT and BV2-KO cell lines by differential ultracentrifugation (namely, NT-Exos and KO-Exos). Transmission electron microscopy (TEM), nanoparticle tracking analysis (NTA), and western blotting analyses were performed to confirm exosomes quality. Similar to previous studies, NT-Exos and KO-Exos exhibited a cup- or sphere-shaped morphology with double-membrane structures (Figure 2A). NTA of the exosomes indicated a range of diameters at 50-150 nm. (Figure 2B). Western blotting analyses also indicated the presence of exosomal markers, including CD63 and TSG101. Moreover, GRP94 and β-Tubulin showed absent or low expression in exosome lysates (Figure 2C).

Scratch wound healing and tube formation assays of HUVEC were performed to detect the functional influence of the above microglia-derived exosomes. Our data indicated that the migration rate of the HUVEC incubated with KO-Exos was significantly higher than that of the HUVEC treated with NT-Exos (Figure 2D-E). Consistently, the statistical analysis of junctions and tube length of HUVEC, as well as tube formation, demonstrated that the KO-Exos group had higher values than the NT-Exos group (Figure 2F-H). To further observe the endocytosis and morphological changes in the recipient cells, we cocultured PKH26-labelled KO-Exos and NT-Exos with HUVEC. Both KO-Exos and NT-Exos could be efficiently taken up by HUVEC, and the staining of VE-cadherin suggests that NT-Exos increase the cell junctions of HUVEC, thus inhibiting cell migration (Figure 2I-J). Thus, these data demonstrated that Tsp-1 in microglia regulates EC behaviour via exosomes, and the loss of Tsp-1 undermines the antiangiogenic features of microglia-derived exosomes.

### Tsp-1 knockout affects the differential profile of miRNA expression in KO-Exos and NT-Exos

**Figure 3:**
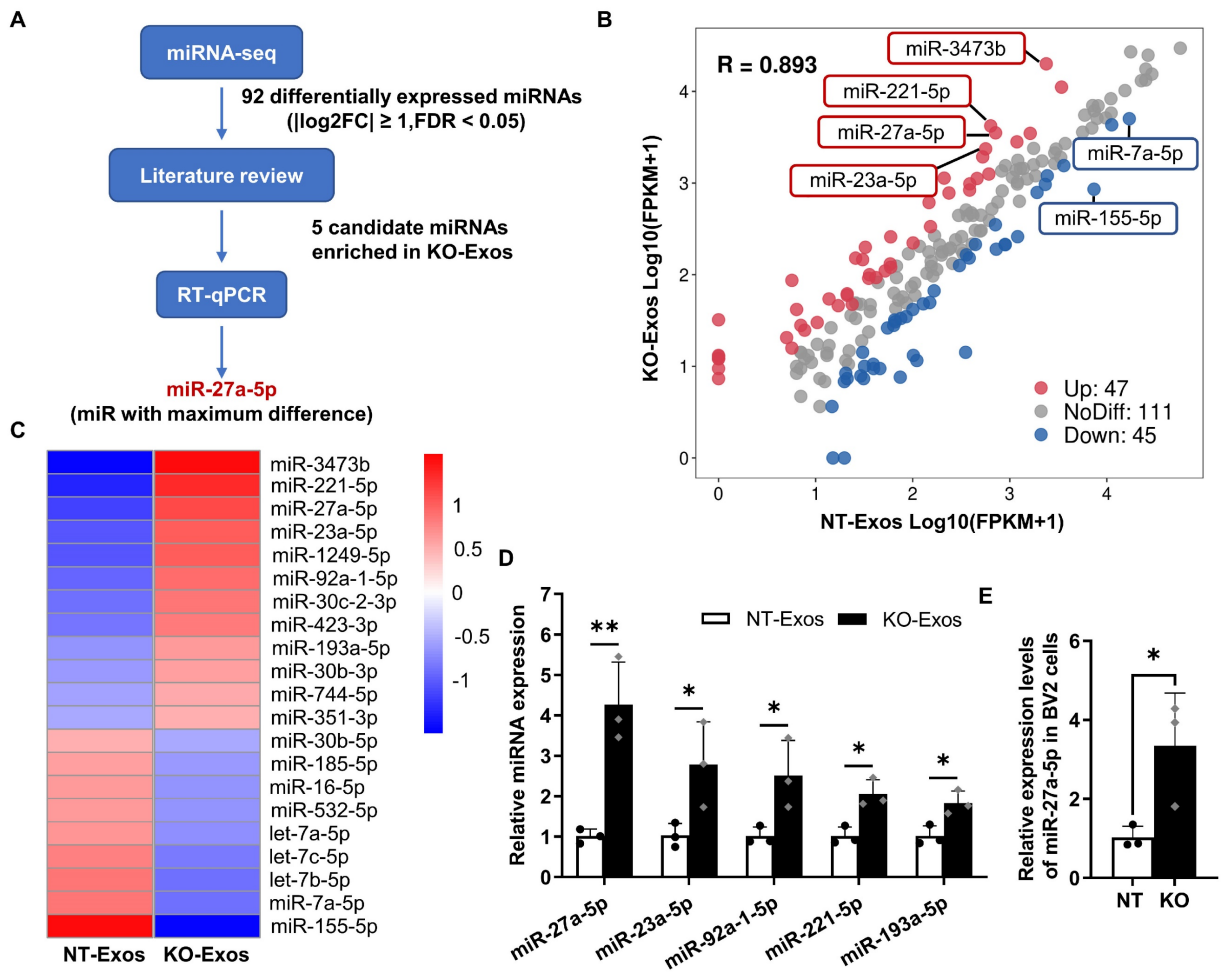
**Differential expression profile of miRNAs in exosomes derived from Tsp-1 KO and NT microglial cell lines. (A)** Schematic diagram of microRNAs (miRNAs) screening and selection.** (B)** Scatter plot illustrating differentially expressed miRNAs in exosomes isolated from the NT or KO BV2 cells. **(C)** The heatmap shows the enriched and significantly variable miRNAs between NT-Exos and KO-Exos (|log2FC| ≥ 1, FDR < 0.05). Red and blue: miRNAs with higher or lower expression respectively. **(D)** Quantitative real-time PCR revealed the relative expression levels of miR-27a-5p, miR-23a-5p, miR-92a-1-5p, miR-211-5p, and miR-193a-5p in NT-Exos and KO-Exos. **P < 0.01, *P < 0.05. n = 3.** (E)** Quantitative real-time PCR revealed the relative expression levels of miR-27a-5p in the NT and KO BV2 cells. *P < 0.05. n = 3. Results are expressed as mean ± SD.

Exosomal miRNAs play an essential role as functional regulators in intercellular interactions. To determine the mechanism of KO-Exos-mediated changes in HUVEC migration, we conducted high-throughput miRNA sequencing (miRNA-seq) to profile miRNAs in KO-Exos and NT-Exos (Figure 3A). A total of 203 mature miRNAs were detected in both KO-Exos and NT-Exos and then described in scattergrams, with 47 miRNAs enriched in KO-Exos and 45 miRNAs depleted (Figure 3B). The top 21 miRNAs with the highest abundance (FDR < 0.05, KO-Exos/NT-Exos ≥ 2 or ≤ 0.5) were chosen to generate a tree with a clear distinction between the two groups. Compared to NT-Exos, KO-Exos showed enrichment of 12 mature miRNAs (Figure 3C). According to some literature review 33-36, five candidate miRNAs (miR-211-5p, miR-27a-5p, miR-23a-5p, miR-92a-1-5p, and miR-193a-5p) were found to be closely related to angiogenic pathways.

Consistent with the miRNA-seq results, RT-qPCR showed that miR-27a-5p, miR-23a-5p, miR-92a-1-5p, miR-211-5p, and miR-193a-5p were enriched in KO-Exos compared to NT-Exos, especially miR-27a-5p (Figure 3D). We further found that the cellular miR-27a-5p level was increased in BV2-KO cells, thereby causing an increase in miRNA levels in exosomes, which may ultimately influence the function of HUVEC (Figure 3E). Moreover, miR-27a-5p was reported to regulate cell migration and proliferation 34,37,38. Thus, we focused on the potential function of exosomal miR-27a-5p in subsequent experiments.

### Deficiency of Tsp-1 leads to the enrichment of miR-27a-5p in KO-Exos and triggers HUVEC migration

**Figure 4:**
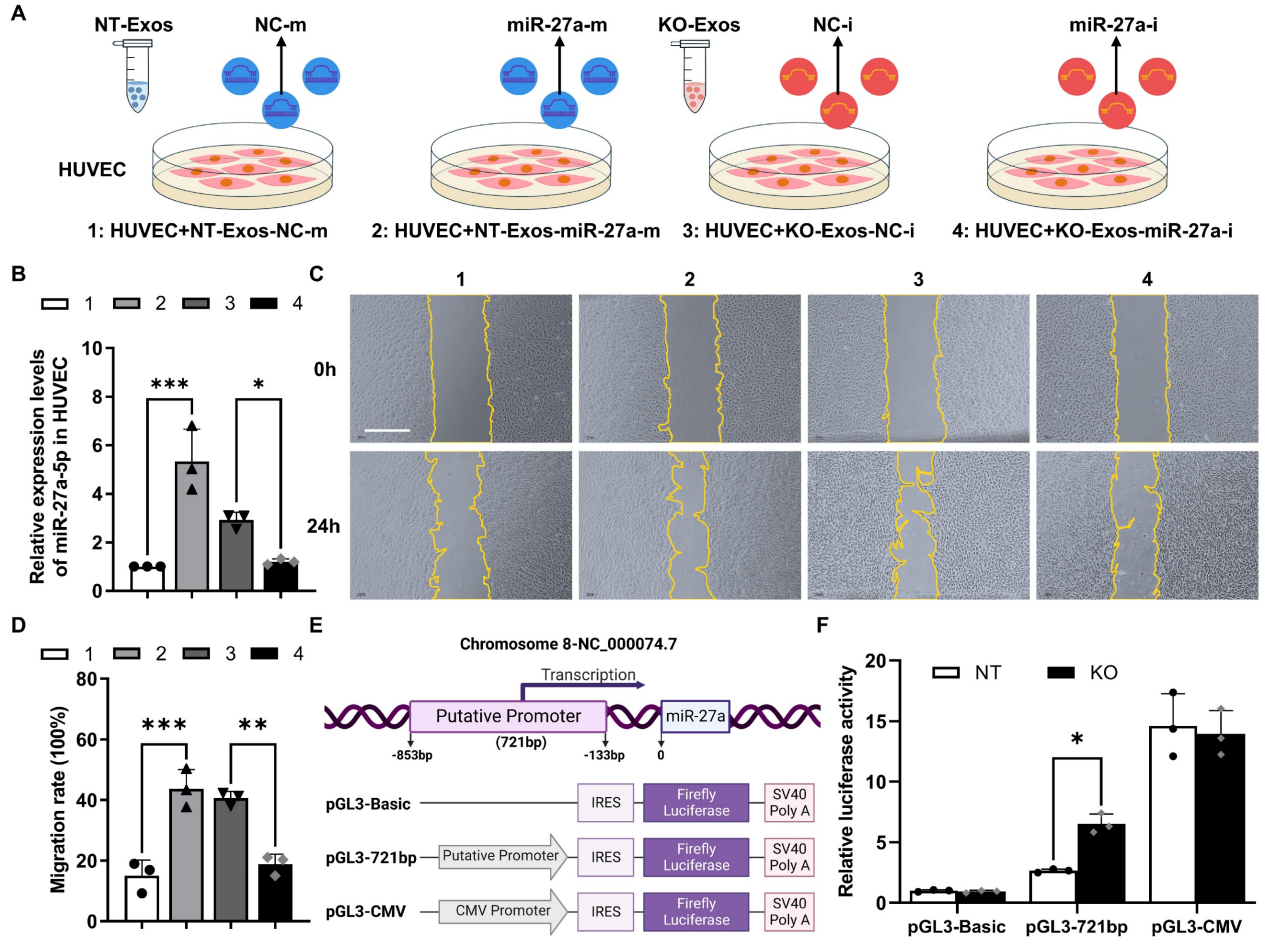
** Exosomal miR-27a-5p from a microglial cell line induces HUVEC migration, and loss of Tsp-1 promotes the transcription of miR-27a-5p. (A)** Schematic diagram of miR-27a-5p gain/loss-of-function experiments design. NT-Exos transfected with synthetic mimic control (NT-Exos-NC-m) or miR-27a-5p mimic (NT-Exos-miR-27a-m), KO-Exos transfected with synthetic inhibitor control (KO-Exos-NC-i) or miR-27a-5p inhibitor (KO-Exos-miR-27a-i), were incubated with HUVEC. **(B)** Quantitative real-time PCR revealed the relative expression level of miR-27a-5p was increased in the HUVEC incubated with NT-Exos-miR-27a-m and decreased in the HUVEC incubated with KO-Exos-miR-27a-i. ***P < 0.001, *P < 0.05. n = 3. Representative images **(C)** and relative quantification **(D)** of wound-healing assays. Incubation of the HUVEC with NT-Exos-NC-m or NT-Exos-miR-27a-m at 0 h and 24 h post-scratch showed that miR-27a-5p overexpression in NT-Exos enhanced the migration of the HUVEC. And the incubation with KO-Exos-NC-i or KO-Exos-miR-27a-i at 0 h and 24 h post-scratch suggested that miR-27a-5p knockdown in KO-Exos could inhibit the migration of HUVEC. Scale bar: 500 μm. ***P < 0.001, **P < 0.01. n = 3. **(E)** Diagram illustrate the putative promoter region of the miR-27a gene and the construction of miR-27a promoter in pGL3-IRES-Luc-reporter. pGL3-Basic was included as a negative control. pGL3-CMV was included as a positive control. **(F)** Relative luciferase activities showed that the pGL3-721bp transcriptional activity was enhanced in BV2-KO cells. *P < 0.05. n = 3. Results are expressed as mean ± SD.

To demonstrate the function of miR-27a-5p in BV2 exosomes, we cocultured HUVEC with exosomes transfected with synthesized miRNA mimic/inhibitor/negative control (NC) (Figure 4A). RT-qPCR detected the expression of miR-27a-5p in HUVEC and showed highly efficient transfer of the synthesized miR-27a-5p mimic from exosomes to recipient cells, while the inhibitor significantly decreased the miR-27a-5p level (Figure 4B). Moreover, a wound healing assay indicated that NT-Exos loaded with a miR-27a-5p mimic significantly increased HUVEC migration. In contrast, KO-Exos transfected with a miR-27a-5p inhibitor strongly inhibited HUVEC migration, which was consistent with the results for NT-Exos (Figure 4C-D). These results suggested that miR-27a-5p was loaded into BV2-derived exosomes and successfully transferred to the recipient cell, thereby contributing to the regulation of cell migration.

Moreover, we monitored the transcriptional activity change of the miR-27a-5p promoter. Based on the specific region containing the promoter of the human miR-27a gene 29**,** we constructed a reporter plasmid to identify the corresponding region of the mouse miR-27a gene. The core sequence of the promoter between -853 and -133 bp was inserted into pGL3-Basic-IRES (Addgene) (Figure 4E). Specifically, pGL3-Basic, pGL3-721bp, and pGL3-CMV were transfected into BV2-NT or BV2-KO cells, and luciferase activity assays demonstrated that pGL3-721bp showed significant active transcription in BV2-KO cells (Figure 4F). Thus, we confirmed that Tsp-1 negatively regulated the expression of miR-27a-5p.

### Smad3 serves as a functional target gene of miR-27a-5p to participate in the migratory activity regulation of exosome recipient cells

**Figure 5:**
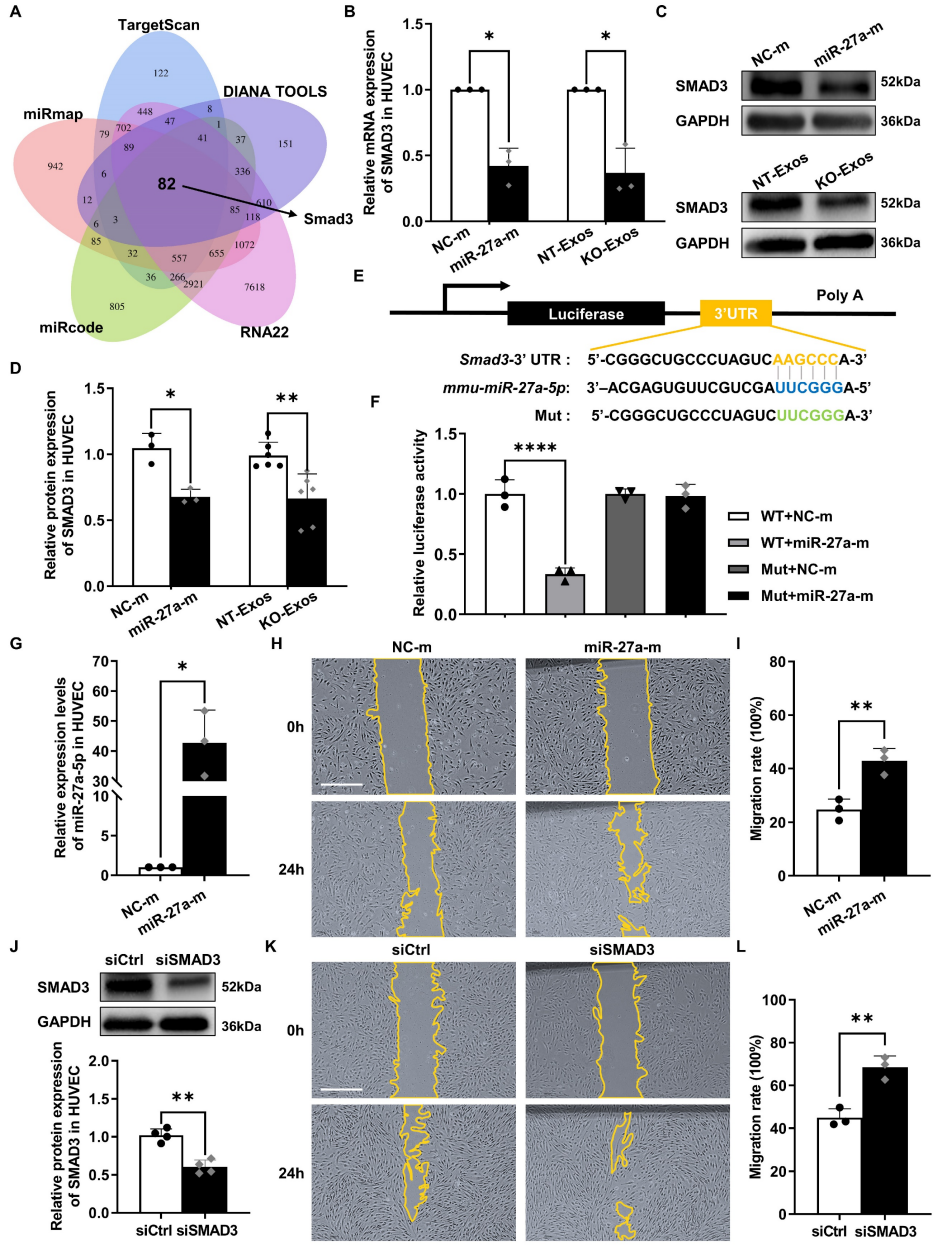
** Smad3 is an effective functional target gene of exosomal miR-27a-5p derived from a microglial cell line. (A)** Venn diagram showing an overlap of the candidate target genes from databases of TargetScan, DIANA TOOLS, RNA22, miRcode, and miRmap. Smad3 is one of the potential target genes of mmu-miR-27a-5p.** (B)** HUVEC were transfected with synthetic mimic control (NC-m) or miR-27a-5p mimic (miR-27a-m), or treated with NT-Exos or KO-Exos. Quantitative real-time PCR revealed that the mRNA level of SMAD3 was decreased in the HUVEC transfected with miR-27a-m or treated with KO-Exos. *P < 0.05. n = 3. Western blot analysis **(C)** and relative quantification of protein expression **(D)** indicated that overexpression of miR-27a-5p (n = 3) or KO-Exos treatment (n = 6) could downregulate SMAD3 expression in HUVEC. **P < 0.01, *P < 0.05. **(E)** Schematic representation of a predicted binding site of mmu-miR-27a-5p in the 3'-UTR of Smad3 mRNA. **(F)** Luciferase reporter assays of the HEK-293T cells cotransfected with NC-m or miR-27a-m and a luciferase reporter containing wild-type or mutant Smad3 3'-UTR. ****p < 0.0001. n = 3. **(G)** HUVEC were transfected with NC-m or miR-27a-m. Quantitative real-time PCR revealed the relative expression level of miR-27a-5p was markedly increased. *P < 0.05. n = 3. Representative images **(H)** and relative quantification **(I)** of the wound-healing assay of the HUVEC transfected with NC-m or miR-27a-m at 0 h and 24 h post-scratch showed that miR-27a-5p overexpression enhanced the migration of the HUVEC. Scale bar: 500 μm.**P < 0.01. n = 3.** (J)** HUVEC were transfected with nonspecific control siRNA (siCtrl) or small interfering RNA against SMAD3 (siSMAD3). siSMAD3 silenced the majority expression of SMAD3 in HUVEC. **P < 0.01. n = 4. Representative images **(K)** and relative quantification **(L)** of the wound-healing assay of the HUVEC transfected with siCtrl or siSMAD3 at 0 h and 24 h post-scratch suggested that SMAD3 knockdown induced a higher migration rate of the HUVEC. Scale bar: 500 μm. **P < 0.01. n = 3. Results are expressed as mean ± SD.

We further explored the target gene of miR-27a-5p. Five public bioinformatics databases (TargetScan, DIANA TOOLS, RNA22, miRcode, miRmap) were used to select candidate genes, and a total of 82 genes overlapped in all five databases (Figure 5A). GO analysis identified the candidate genes associated with cell growth and cell junction regulation (Figure S2A). Then, we confirmed the changes in mRNA levels of highly related genes, such as Smad3, Smad4, Bdnf, Sfrp1, Notch2, Tgfbr3, and Egfr, in HUVEC after transfection with the miR-27a-5p mimic (Figure 5B, S2B). Moreover, based on some previous investigations 39-41, we focused on the candidate gene Smad3, a member of the Smad family, which is a transcription factor highly related to the regulation of cell migration and proliferation. We then verified the connection between Smad3 and miR-27a-5p. Both the mRNA level (Figure 5B) and protein level (Figure 5C-D) of Smad3 were decreased after transfection of the miR-27a-5p mimic into HUVEC. This finding was consistent with the results of exosomes cocultured with recipient cells, in which Smad3 downregulation was observed in the KO-Exos incubation group versus that of NT-Exos (Figure 5B-D).

Moreover, we identified the interaction between miR-27a-5p and Smad3 mRNA through a dual-luciferase reporter assay. This experiment was performed by cloning the predicted interaction site of the Smad3 3'UTR sequence (wild-type and mutant) into the 3'UTR of the luciferase gene on the reporter plasmid (Figure 5E). As expected, the miR-27a-5p mimic significantly decreased the luciferase activity (Figure 5F), which indicated a strong interaction between miR-27a-5p and the 3'UTR target sequence of Smad3.

Furthermore, we confirmed that Smad3 is the primary target gene of the miR-27a-5p-mediated function in migratory activity regulation. We compared the functional effect on cell migration between miR-27a-5p and a specific Smad3-targeting siRNA, and the results showed that both are equally effective in promoting cell migration (Figure 5G-L). These results further demonstrated that Smad3 acted as the primary target gene of miR-27a-5p, which played a key role in the regulation of cell migration.

### NT-Exos and KO-Exos diffuse in the retina and are potentially absorbed by vascular ECs thereby leading to different destinies of retinal neovascularization in mice with OIR

**Figure 6:**
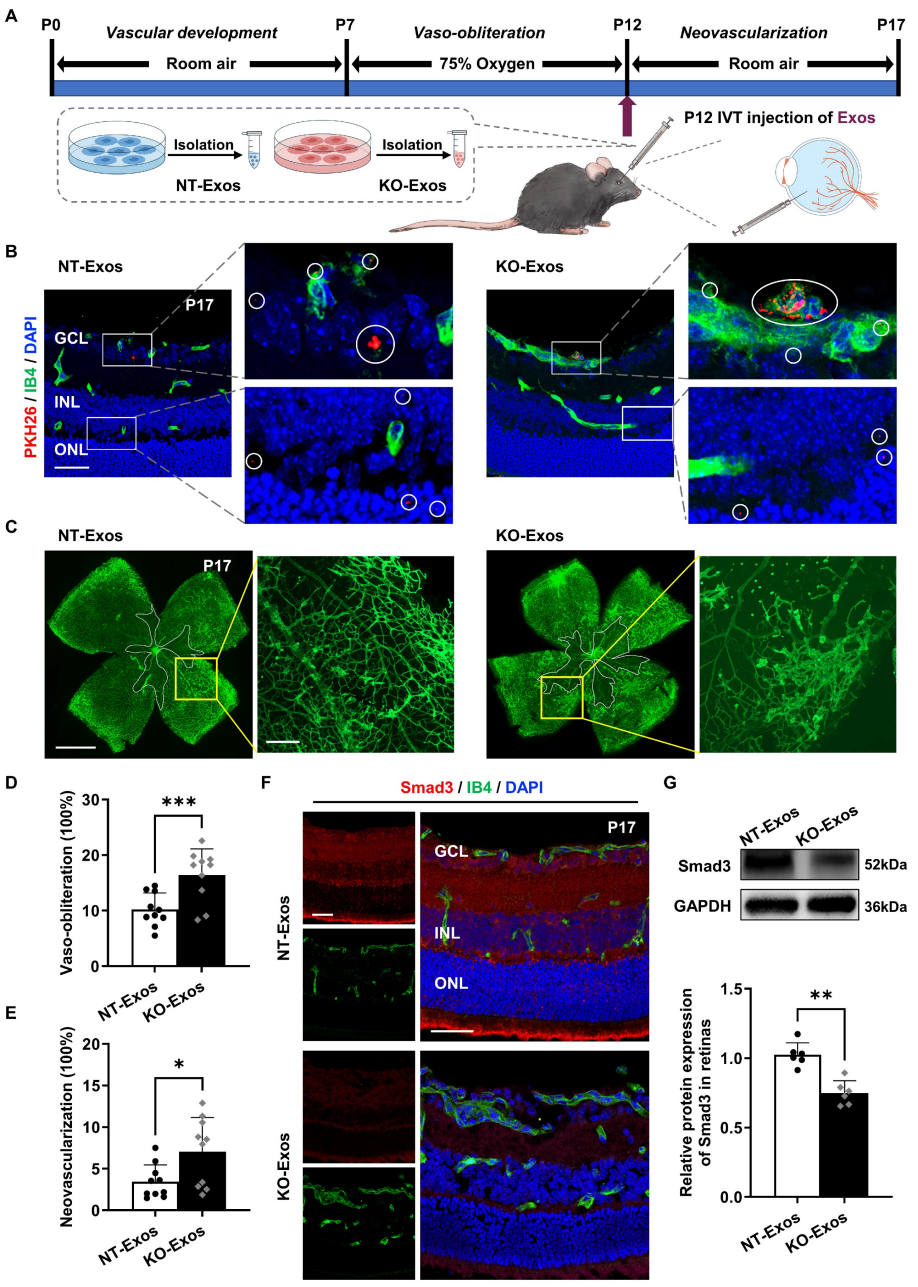
***In vivo* tracking of NT-Exos and KO-Exos and corresponding observation of retinal angiogenesis in mice with OIR. (A)** Schematic illustration of the mice with oxygen-induced retinopathy (OIR) receiving intravitreal (IVT) injection of exosomes. **(B)** Representative confocal images of retinal sections derived from OIR mice treated with the PKH26-labelled NT-Exos or KO-Exos. Exosomes were taken up by multiple cell layers of the retina (enlarged white boxes; red dots within white circles). Co-staining of PKH26 and Isolectin B4 (IB4) exhibited that some exosomes internalized into the retinal vascular endothelial cells (ECs). (enlarged white boxes; upper panel). PKH26, red; IB4, green; DAPI, blue. Scale bar: 50μm. n = 6 mice/group with three litters examined. **(C)** Representative images of IB4-stained retinal flat-mount at P17 from the OIR mice treated with NT-Exos and KO-Exos. Vaso-obliterated blood area in the centre was divided (within the white borders), while the neovascularization areas were enlarged (yellow boxes). Scale bar: 1 mm (left panel); 200μm (right panel). Quantification of vaso-obliteration **(D)** and neovascularization **(E)** in OIR retinas was expressed as a percentage of total retinal areas. ***P < 0.001, *P < 0.05. n = 10 mice/group with three litters examined. **(F)** Representative confocal images of Smad3 and IB4 co-staining in retinal sections from mice with OIR treated with NT-Exos and KO-Exos then sacrificed at P17. DAPI was used to label the different retinal layers (Smad3, red; IB4, green; DAPI, blue). Scale bar:50μm. n = 8 mice/group with three litters examined. **(G)** Western blot analysis and relative quantification of protein expression exhibited that KO-Exos treatment could downregulate the Smad3 expression in the OIR retinas at P17. **P < 0.01. n = 6 mice/group with three litters examined. Results are expressed as mean ± SD. *GCL*: ganglion cell layer, *INL*: inner nuclear layer, *ONL*: outer nuclear layer.

We performed *in vivo* assays in the OIR animal model to further validate the *in vitro* results. We initially confirmed the ability of BV2-derived exosomes to infiltrate the retina by labelling them with PKH26 and administering the intravitreal injection. As shown in Figure 6A, labelled exosomes (NT-Exos and KO-Exos) from two different BV2 cell lines were intravitreally injected into the left eye of mice with OIR at P12. Immunofluorescence staining at P17 showed that both NT-Exos and KO-Exos were distributed throughout the retinal cell layers. Besides, co-staining of PKH26 and Isolectin B4 (IB4) indicated that some exosomes internalized into the retinal vascular ECs (Figure 6B). Then, we performed two additional groups of injections at P12 using NT-Exos and KO-Exos without PKH26 to validate their angiogenic influences. The vaso-obliteration (VO) and neovascularization (NV) areas of the retina were analysed at P17. KO-Exos significantly increased the NV area and VO area in the retina compared to those in the NT-Exos-treated group (Figure 6C-E). Moreover, the expression of the candidate functional protein Smad3 decreased in the KO-Exos-treated retinas according to immunofluorescence staining and western blotting (Figure 6F-G). The staining of IB4 also revealed that more blood vessels formed in the KO-Exos group (Figure 6F). Together, it can be concluded that NT-Exos/KO-Exos possess the capability to penetrate different layers of the retina and are partially absorbed by the vascular ECs. This is in line with the observed phenotype of Smad3 suppression across retinal layers following KO-Exos treatment, as well as the concurrent increase of abnormal neovascularization in the retina of mice with OIR.

### KO-Exos with miR-27a-5p knockdown regain the ability to inhibit angiogenesis in the retina of mice with OIR by maintaining retinal Smad3 expression

**Figure 7:**
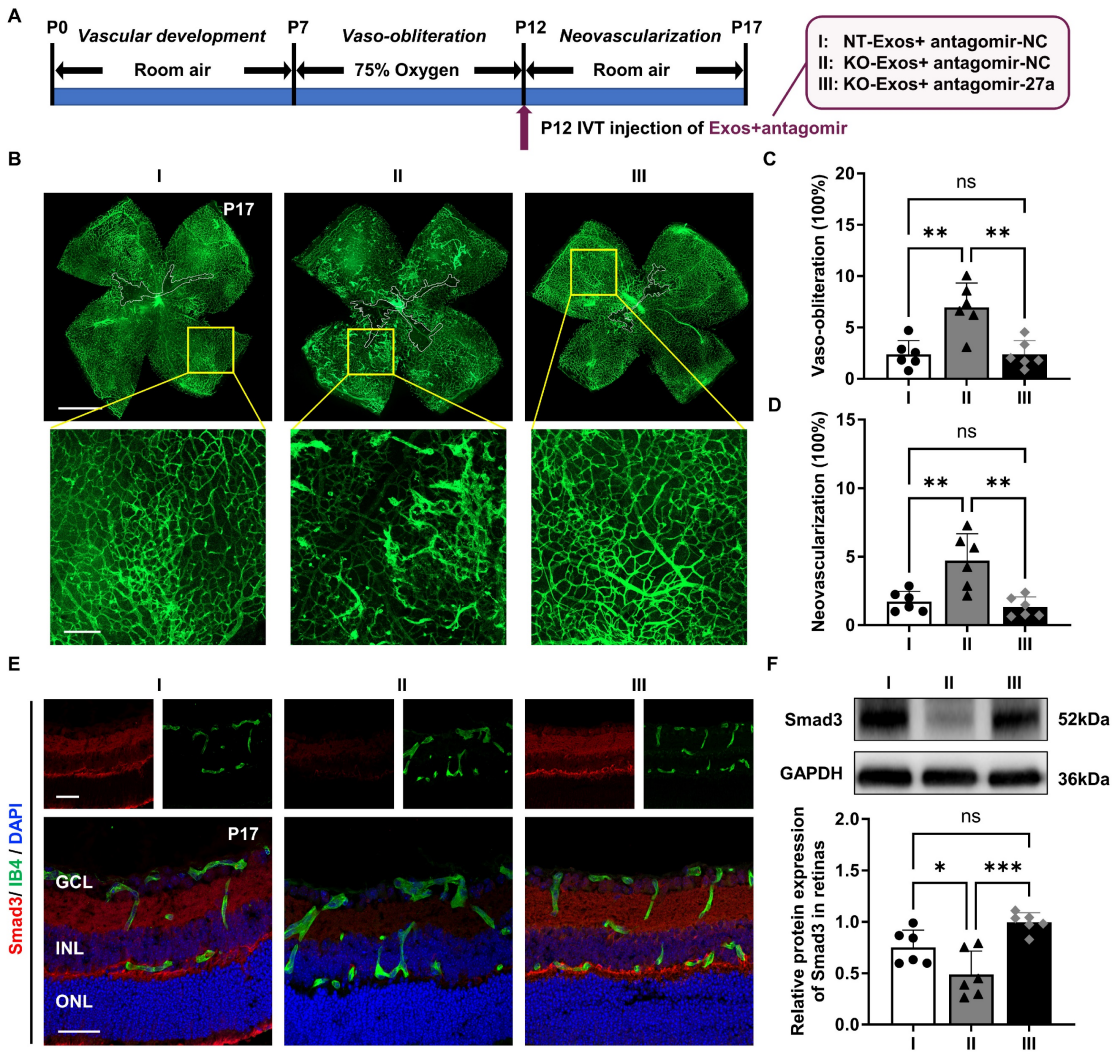
***In vivo* validation of miR-27a-5p in KO-Exos that confirms its impact on Smad3, retinal vaso-obliteration and neovascularization in mice with OIR. (A)** Schematic representation of the mice with oxygen-induced retinopathy (OIR) treated with intravitreal (IVT) injection of exosomes loaded with antagomir. I: NT-Exos+antagomir-NC; II: KO-Exos+antagomir-NC; III: KO-Exos+antagomir-27a. **(B)** Representative images of retinal flat-mount at P17 stained with Isolectin B4 (IB4) were obtained from the mice with OIR treated with exosomes loaded with antagomir. Vaso-obliterated blood area in the centre was divided (within the white borders), while the neovascularization areas were enlarged (yellow boxes). Scale bar: 1 mm (upper panel). Scale bar: 200μm (lower panel). Quantification of vaso-obliteration **(C)** and neovascularization **(D)** in OIR retinas was expressed as a percentage of total retinal areas. **P < 0.01, ns: non-significant. n = 6 mice/group with three litters examined. **(E)** Representative confocal images of retinal sections co-stained with Smad3 and IB4 from the mice with OIR treated with exosomes loaded with antagomir. DAPI exhibits the retinal layers (Smad3, red; IB4, green; DAPI, blue). Scale bar:50μm. n = 6 mice/group with three litters examined. **(F)** Western blot analysis and relative quantification of Smad3 protein expression in the OIR retinas at P17. ***P < 0.001, *P < 0.05, ns: non-significant. n = 6 mice/group with three litters examined. Results are expressed as mean ± SD. *GCL*: ganglion cell layer, *INL*: inner nuclear layer, *ONL*: outer nuclear layer.

Based on the evidence *in vitro*, the Tsp-1 of microglia inhibits miR-27a-5p expression, and we hypothesize that KO-Exos with increased miR-27a-5p could inhibit Smad3 expression of retinal ECs and promote angiogenesis in mice with OIR. To prove the pathway* in vivo*, we employed miR-27a-5p antagomir (antagomir-27a) to suppress the expression of miR-27a-5p in KO-Exos prior to intravitreal injection of mice with OIR. As shown in Figure 7A, Exos were transfected with antagomir and injected at P12 during OIR modelling. At P17, the retinal flat-mount staining of Isolectin B4 revealed that miR-27a-5p antagomir rescued the antiangiogenic effect of KO-Exos to a level compatible with NT-Exos (Figure 7B-D). Moreover, a consistent expression trend of the target gene Smad3 was revealed by immunofluorescence analysis of the retinal frozen sections and western blotting. Specifically, Smad3 was significantly inhibited in the KO-Exos group treated with antagomir-NC and recovered to a high level in the KO-Exos group treated with antagomir-27a, which was compatible with the group of antagomir-NC treated NT-Exos (Figure 7E-F).

Collectively, these* in vivo* results of the OIR animal model provide strong evidence for the involvement of the Tsp-1/miR-27a-5p/Smad3 axis in retinal angiogenesis. Tsp-1 in activated microglia plays an important role in retinal neovascularization, and this function is executed by microglial exosomes with an essential component, miR-27a-5p, which regulates the expression of Smad3 in recipient vascular ECs and then defines the destiny of retinal neovascularization.

## Discussion

Microglia show rapid responses to injury and changes in the microenvironment and are expected to regulate angiogenesis in the retina. Microglia with beneficial effects in the regulation of angiogenesis urgently need to be explored and utilized. In addition, bioinformatic analyses of scRNA-seq data help elucidate the antiangiogenic features of microglia 17. In the present study, we found that a large group of microglia, MG3, demonstrated strong expression of genes related to wound healing and antiangiogenesis in scar-free spinal cord repair. Tsp-1 was defined as a strong marker gene of this subtype of microglia (Figure 1A-C). However, from a similar microglia subtype in the retina, RMG1, those cells with strong Tsp-1 expression only account for a small number of total microglia (Figure 1D-F). Consistently, our experiments indicate that the slight increase in Tsp-1^+^ microglia during OIR modelling cannot rescue the progression of neovascularization (Figure 1G-H). This finding is in accordance with the fact that many studies have defined the main phenotype that microglia contribute to in retinal pathological angiogenesis as detrimental 14-16. In general, our discovery indicates a potential beneficial effect of Tsp-1 in microglia-mediated angiogenic regulation.

Secondly, our study showed that intercellular communication between Tsp-1^+^ microglia and ECs was undertaken by exosomes. Previous studies have demonstrated that exosomes are prioritized as a collection of essential cell-derived components that dominate intercellular communication 22, 23. As expected, our data also indicated that NT-Exos from the Tsp-1^+^ microglial cell line significantly inhibited the adhesion, proliferation, migration, and tube formation of HUVEC. In contrast, KO-Exos did not show antiangiogenic bioactivity (Figure 2). Moreover, these results were confirmed *in vivo* (Figure 6C-F). Therefore, we conclude that microglial Tsp-1 could regulate retinal angiogenesis via exosomes.

Thirdly, we elucidated the antiangiogenic pathway regulated by Tsp-1 and mediated by the transfer of exosomes derived from microglia to ECs. Exosomes are critical in delivering miRNAs as specific inhibitors of recipient cell gene expression via an RNA-induced silencing complex that triggers mRNA degradation 20,21,42. Our miRNA-seq results indicated that the loss of Tsp-1 leads to the enrichment of miR-27a-5p in KO-Exos (Figure 3). Moreover, Tsp-1 inhibited the active region of the miR-27a-5p promoter to downregulate its expression in exosomes (Figure 4E-F). Several previous studies indirectly supported our discovery. For instance, miR-27a-5p has been demonstrated to be upregulated and potentially promote angiogenesis in the retina of a diabetic retinopathy mouse model 37. miR-27a-5p alleviated cell apoptosis in a mouse liver ischaemia‒reperfusion model 38. Thus, the evidence indicates that exosomal miR-27a-5p could be negatively regulated by Tsp-1 to maximize the antiangiogenic function of NT-Exos.

Finally, we further explored the target genes of miR-27a-5p and found that Smad3 is an essential candidate gene of recipient ECs that affects angiogenesis 43-45. Smad3 is a receptor-activated Smad belonging to the Smad protein family. Unlike common mediator Smads and inhibitory Smads, it tends to directly bind a region of promoter and affect the downstream gene expression 46-48. Numerous studies have revealed the role of Smad3 in the maintenance of vascular homeostasis. Activated Smad3 can attenuate the MAPK/ERK pathway by inhibiting the phosphorylation of both mitogen-activated protein kinases and extracellular signal-regulated kinase-1/2, consequently restricting the growth of retinal endothelial cells 39. During vascular development, the expression of Smad3 prevents abnormal angiogenesis by maintaining the expression of N-cadherin and sphingosine-1-phosphate receptor-1 40. Moreover, Smad3 cooperates with Runx1 to maintain the differentiation state of the corneal epithelium rather than proliferation 41. Consistently, our results validated the inhibition of Smad3 by miR-27a-5p at both the mRNA and protein levels (Figure 5B-D). Furthermore, we confirmed the inhibition of retinal Smad3 by KO-Exos and the intensive inhibition of retinal neovascularization by NT-Exos *in vivo* (Figure 6C-G). Besides, the neovascularization inhibitory capacity of KO-Exos was restored to a level compatible with that of NT-Exos through the inhibition of miR-27a-5p using antagomir (Figure 7B-F). Together, our study reveals a clear molecular pathway by which the Tsp-1/miR-27a-5p/Smad3 axis is involved in microglia-related and exosome-mediated antiangiogenic regulation of the retina.

In summary, our study demonstrates that Tsp-1 from a unique subtype of retinal microglia modulates cell-to-cell communication between microglia and vascular endothelial cells. Tsp-1 not only directly intervenes in the migration and proliferation of ECs but also alters the abundance of some microRNAs in exosomes, which facilitates the antiangiogenic function of Tsp-1. It is elucidated that Tsp-1 inhibits the transcriptional activity of miR-27a-5p in the microglia to decrease its abundance in exosomes, thereby reducing the inhibition of the target gene Smad3, which limits vascular overgrowth in the retina. Therefore, the study reveals a novel mechanism for the involvement of microglial Tsp-1 in antiangiogenesis, which could be extended as a potential clinical therapy. Tsp-1 is a well-known negative regulator of angiogenesis and is expressed in various ocular cell types, including endothelial cells, pericytes, astrocytes, and microglia 49-51. However, broadly repressing or augmenting Tsp-1 in the retina as a therapeutic strategy is not feasible due to its diversified function in individual cell types 50. Here, the identification of retinal Tsp-1^+^ microglia with specific properties in angiogenesis regulation could be utilized to achieve precision therapeutics, by which engineering the expression of Tsp-1 and miR-27a-5p in microglia to produce exosomes with maximized anti-angiogenic potency as an innovative and precise therapeutic approach.

## Supplementary Material

Supplementary figures and tables.

## Abbreviations

antagomir-27a: miR-27a-5p antagomir
ECs: endothelial cells
Exos: exosomes
FC: fold change
FDR: false discovery rate
GO: gene ontology
HUVEC: human umbilical vein endothelial cells
Iba-1: induction of brown adipocytes 1
IB4: isolectin B4
IVT: intravitreal
KO: knockout
MG: microglia
miR: microRNA
miRNA-seq: miRNA sequencing
miR-27a-i: miR-27a-5p inhibitor
miR-27a-m: miR-27a-5p mimic
Mut: mutant
NC: negative control
NC-i: synthetic inhibitor control
NC-m: synthetic mimic control
NT: negative target
NV: neovascularization
OIR: oxygen-induced retinopathy
RMG: retinal microglia
scRNA-seq: single-cell RNA sequencing
sgRNA: single-guided RNA
Smad3: SMAD family member 3
Tsp-1: thrombospondin-1
VEGF: vascular endothelial growth factor
VO: vaso-obliteration
WT: wild-type
