# Supplementary material for: Tsp-1+ microglia attenuate retinal neovascularization by maintaining the expression of Smad3 in endothelial cells through exosomes with decreased miR-27a-5p
Source: Theranostics. 2023 Jun 26;13(11):3689–706. doi: 10.7150/thno.84236 (PMC10334831; doi:10.7150/thno.84236)
Supplement: Supplementary file 1 — Supplementary figures and tables. [file thnov13p3689s1.pdf]

Tsp-1^{+} microglia attenuate retinal neovascularization by maintaining the expression of Smad3 in endothelial cells through exosomes with decreased miR-27a-5p

Supplementary Figures S1-S2 and Tables S1-S3

Qian Luo[1,2#], Zihua Jiang[1,2#], Jingyi Jiang[1,2], Linxi Wan[1,2], Yan Li[1,2], Yuke Huang[1,2], Jin Qiu[1,2], Keming Yu[1,2], Jing Zhuang[1,2*]*

1. State Key Laboratory of Ophthalmology, Zhongshan Ophthalmic Center, Sun Yat-sen University, No.7 Jinsui Road, Tianhe District, Guangzhou, 510060, China

2. Guangdong Provincial Key Laboratory of Ophthalmology and Visual Science, Guangzhou, 510060, China

Supplementary Figures

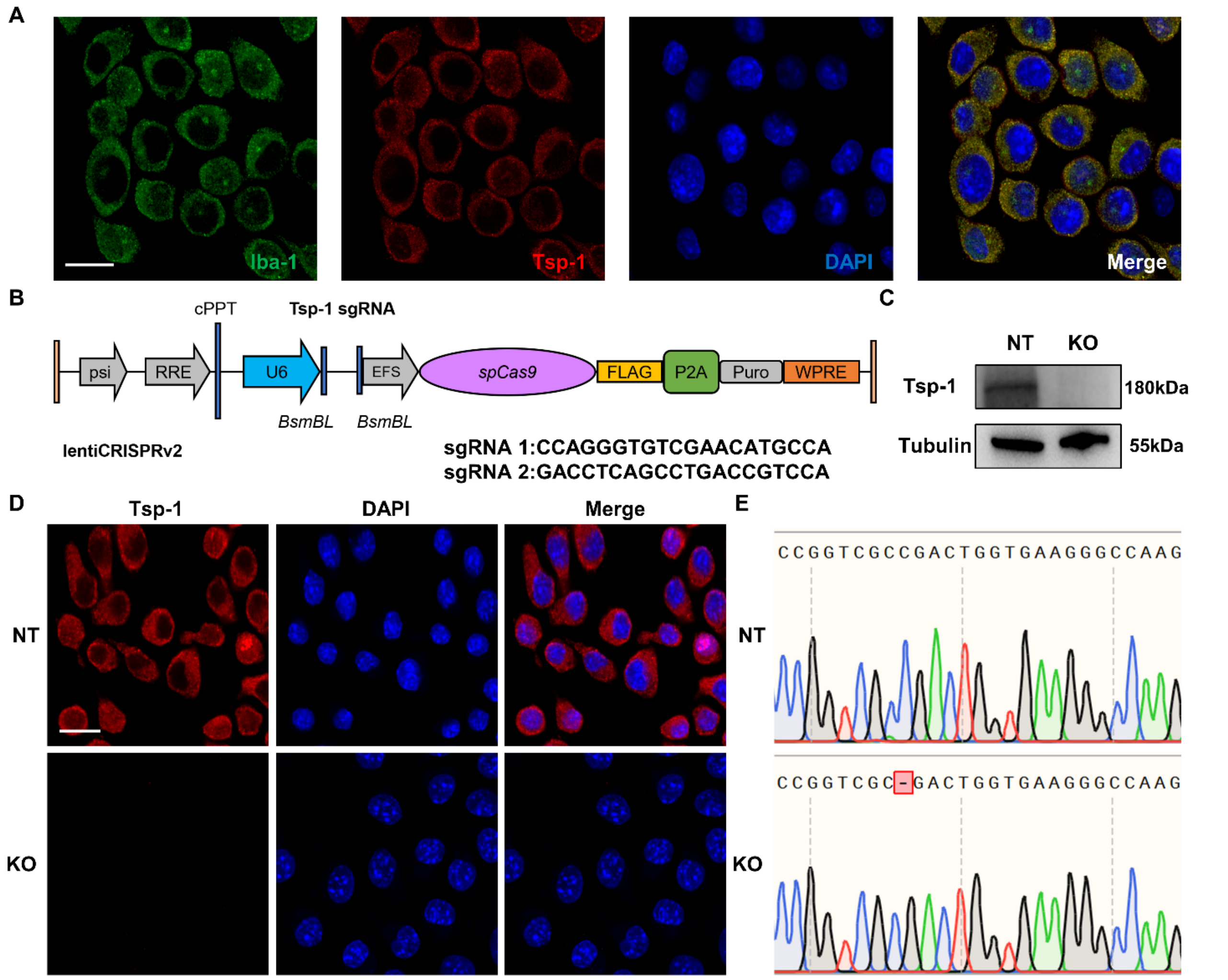

Figure S1. Stable knockout of Tsp-1 in the BV2 microglial cell line. (A) Representative confocal images of double staining for Iba-1 and Tsp-1 in BV2 microglia (Iba-1, green; Tsp-1, red; DAPI, blue). Scale bar: 20 μm. n = 3. **(B)** Schematic diagram of the lentiCRISPRv2 plasmid targeting Tsp-1. Two single-guide RNAs (sgRNAs) were designed to knock out the Tsp-1 gene. Western blot results **(C)** and representative confocal images **(D)** of Tsp-1 expression levels in the negative target (NT) and Tsp-1 knockout (KO) BV2 cells. The BV2-KO cells lacked Tsp-1 expression. (Tsp-1, red; DAPI, blue). Scale bar: 20 μm. n = 3. **(E)** Sanger sequencing of lentivirus-infected microglia, including the BV2-NT and BV2-KO cells. Dashes indicate deleted nucleotides. n = 5.

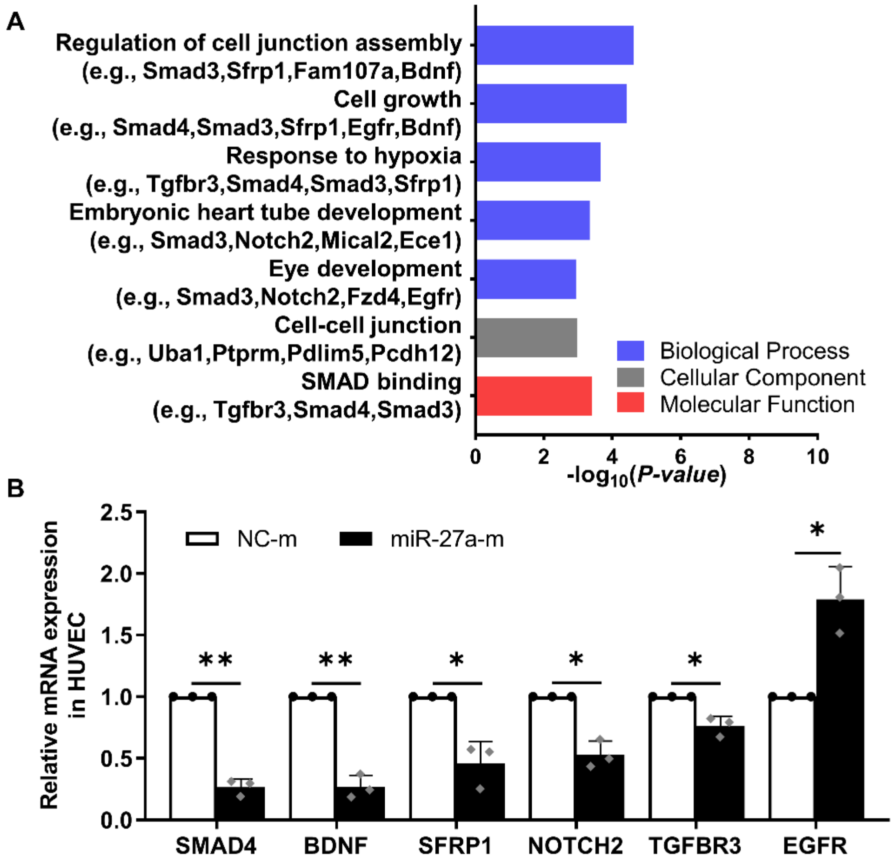

Figure S2. Analysis of miR-27a-5p candidate genes with Gene Ontology enrichment and RT–qPCR. (A) Gene Ontology (GO) analysis of miR-27a-5p candidate target genes. A two-sided statistical test in function enrichGO was used. **(B)** HUVEC were transfected with synthetic mimic control (NC-m) or miR-27a-5p mimic (miR-27a-m), and quantitative real-time PCR revealed the relative mRNA expression levels of SMAD4, BDNF, SFRP1, NOTCH2, TGFBR3, and EGFR. ***P* < 0.01, **P* < 0.05. n = 3. Results are expressed as mean ± SD.

Supplementary Tables

Table S1. RT–qPCR primer sequences

Primer	Sequence (5′–3′)
F-miR-27a-5p	GGCTTAGCTGCTTGTGAGCA
F-miR-23a-5p	GGTTCCTGGGGATGGGATTT
F-miR-221-5p	CACCTGGCATACAATGTAGATTTCTG
F-miR-193a-5p	GGTCTTTGCGGGCAAGATGA
F- miR-92a-1-5p	GGTTGGGATTTGTCGCAATGC
Universal-R	CAGTGCGTGTCGTGGAGT
F-U6	GCTTCGGCAGCACATATACTAAAAT
R-U6	CGCTTCACGAATTTGCGTGTCAT
F-h-SMAD3	TGAGGCTGTCTACCAGTTGACC
R-h-SMAD3	GTGAGGACCTTGTCAAGCCACT
F-h-SMAD4	CTACCAGCACTGCCAACTTTCC
R-h-SMAD4	CCTGATGCTATCTGCAACAGTCC
F-h-BDNF	CATCCGAGGACAAGGTGGCTTG
R-h-BDNF	GCCGAACTTTCTGGTCCTCATC
F-h-SFRP1	CAATGCCACCGAAGCCTCCAAG
R-h-SFRP1	CAAACTCGCTGGCACAGAGATG

F-h-NOTCH2	GTGCCTATGTCCATCTGGATGG
R-h-NOTCH2	AGACACCTGAGTGCTGGCACAA
F-h-TGFBR3	TGGAGTCTCCTCTGAATGGCTG
R-h-TGFBR3	CCATTATCACCTGACTCCAGATC
F-h-EGFR	AACACCCTGGTCTGGAAGTACG
R-h-EGFR	TCGTTGGACAGCCTTCAAGACC
F-h-ACTIN	CACCACACCTTCTACAATGAG
R-h-ACTIN	TAGCACAGCCTGGATAGCAAC

Table S2. miRNA mimic, inhibitor, and antagomir sequences

Name	Sequence (5′–3′)
miR-27a-5p mimic	AGGGCUUAGCUGCUUGUGAGCA
miRNA mimic-NC	UCACAACCUCCUAGAAAGAGUAGA
miR-27a-5p inhibitor	UGCUCACAAGCAGCUAAGCCCU
miRNA inhibitor-NC	UCUACUCUUUCUAGGAGGUUGUGA
miR-27a-5p antagomir	UGCUCACAAGCAGCUAAGCCCU
miRNA antagomir-NC	CAGUACUUUUGUGUAGUACAAA

Table S3. 3′ UTR target sequences of Smad3 for pMIR-report vector cloning

Name	Sequence (5′–3′)
Luc-Smad3-WT-F	AGCTTGCAAACGGGCTGCCCTAGTCAAGCCCAGTCCCTTCAACAGTATGTCTGAT
Luc-Smad3-WT-R	CTAGATCAGACATACTGTTGAAGGGACTGGGCTTGACTAGGGCAGCCCGTTTGCA
Luc-Smad3-Mut-F	AGCTTGCAAACGGGCTGCCCTAGTCTTCGGGTGTCCCTTCAACAGTATGTCTGAT
Luc-Smad3-Mut-R	CTAGATCAGTCATACTGTTGAAGGGACACCCGAAGACTAGGGCAGCCCGTTTGCA
